# The Warburg Effect Suppresses Oxidative Stress Induced Apoptosis in a Yeast Model for Cancer

**DOI:** 10.1371/journal.pone.0004592

**Published:** 2009-02-25

**Authors:** Christoph Ruckenstuhl, Sabrina Büttner, Didac Carmona-Gutierrez, Tobias Eisenberg, Guido Kroemer, Stephan J. Sigrist, Kai-Uwe Fröhlich, Frank Madeo

**Affiliations:** 1 Institute of Molecular Biosciences, University of Graz, Graz, Austria; 2 INSERM, U848, Villejuif, France; 3 Institut Gustave Roussy, Villejuif, France; 4 Université Paris Sud, Paris 11, Villejuif, France; 5 Genetik. Institut für Biologie, FU Berlin, Berlin, Germany; 6 NeuroCure Cluster of Excellence, Charité Berlin, Berlin, Germany; Newcastle University, United Kingdom

## Abstract

**Background:**

Otto Warburg observed that cancer cells are often characterized by intense glycolysis in the presence of oxygen and a concomitant decrease in mitochondrial respiration. Research has mainly focused on a possible connection between increased glycolysis and tumor development whereas decreased respiration has largely been left unattended. Therefore, a causal relation between decreased respiration and tumorigenesis has not been demonstrated.

**Methodology/Principal Findings:**

For this purpose, colonies of *Saccharomyces cerevisiae*, which is suitable for manipulation of mitochondrial respiration and shows mitochondria-mediated cell death, were used as a model. Repression of respiration as well as ROS-scavenging via glutathione inhibited apoptosis and conferred a survival advantage during seeding and early development of this fast proliferating solid cell population. In contrast, enhancement of respiration triggered cell death.

**Conclusion/Significance:**

Thus, the Warburg effect might directly contribute to the initiation of cancer formation - not only by enhanced glycolysis - but also via decreased respiration in the presence of oxygen, which suppresses apoptosis.

## Introduction

In the 1920's Otto Warburg described the most common biochemical phenotype of tumor cells: even in the presence of a normal oxygen level, glycolysis is highly elevated and accompanied by high lactate production as well as drastically reduced mitochondrial respiration [1]–[3]. Though this phenotype is the basis for tumor diagnostics monitoring glucose consumption [4], challenging questions remain elusive: For example how tumor cells adopt this phenotype by different mechanisms [5], [6], whether both phenomena (high glycolysis and decreased respiration) are causally linked [7], [8], and finally their specific contribution to tumorigenesis [7], [9]–[11].

Rapidly proliferating yeasts show similar fermentation values as tumor cells [12], thus allowing to study the relationship between energy metabolism and maximal proliferation. Notably, programmed cell death, which protects against tumorigenesis, is intimately linked to mitochondrial processes in mammalian cells. Yeast cells can also undergo programmed cell death [13], [14] in a mitochondria-dependent manner [15], [16], including cytochrome c and AIF release, channel opening upon human Bax expression [17]–[19], depolarisation of mitochondrial membrane potential [20], and mitochondrial fragmentation [21].

The special ability of Crabtree-positive yeasts like *Saccharomyces cerevisiae* to switch on and off respiration in response to changes in the carbon source and the possibility of producing viable strains lacking mitochondrial DNA (rho^0^), allow the stringent genetic evaluation of the role of mitochondria during cell death [15], [22]. Here, we assess the possible link between suppressed cell death and inhibited oxidative phosphorylation, in colonies grown from a single cell, resembling tumor growth.

## Results and Discussion

### In a maximal proliferating solid cell population, respiration enhances ROS production and apoptosis

**Figure 1:**
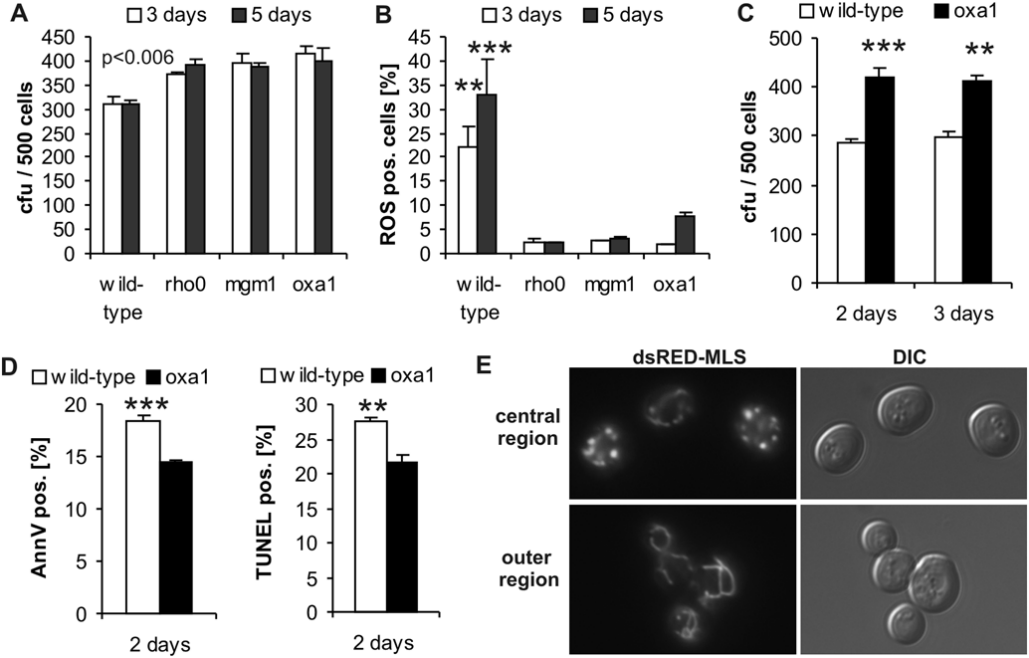
Abrogation of respiration suppresses apoptosis and ROS production in a maximal proliferating solid cell population. (A) Isolated colonies were grown from single cells, manipulated onto agar plates. Clonogenic assay of cells removed from colonies of the wild-type strain, rho^0^ (no mitochondrial DNA) and two single gene-deletion strains (*mgm1* and *oxa1*), grown on SCGlu. After 3 days 500 cells of whole colonies, after 5 days 500 cells of the central colony-region were analysed (mean±SEM, *n* = 2). Statistical significance of p<0.006 compares colony forming units (cfu) of mutant strains to wild-type strain at respective time-points. (B) Cells from the whole colony (3 days) as well as from the central region (5 days) were stained for reactive oxygen species (ROS) with dihydroethidium and analysed by FACSAria flow cytometry (mean±SEM, *n* = 2; **p<0.01, ***p<0.001 compared to deletion strains at respective time-points). (C) Approximately 50 (2 days) and 10 (3 days) colonies grown on SCGlu plates were washed off and clonogenic assays were performed with the collected cells (mean±SEM, *n* = 5; **p<0.01, ***p<0.001). (D) Approximately 50 colonies grown on SCGlu plates for 2 days were stained for phosphatidylserine exposition (AnnV) and DNA breakage (TUNEL) and analyzed by FACSAria flow cytometry (mean±SEM, *n* = 3; **p<0.01, ***p<0.001). (E) Fluorescence microscopy from central and outer region of 5 days old wild-type colony cells, harbouring plasmid dsRED-MLS.

*S. cervevisiae* cells inherit a unique glucose repression system that upon growth on glucose drastically suppresses respiration independently of oxygen availability [23]. Modelling tumor growth, we tested if repressed respiration influences cell-survival during growth of cell populations starting from a single cell on glucose media. Three different strains incapable of respiration together with isogenic wild-type *S. cerevisiae* were used in a colony assay where isolated colonies were grown from single cells. Cells removed from colonies during early development of a rho^0^ strain colony exhibit significantly increased survival compared to the wild-type strain (Fig. 1A). Consistently, the generation of reactive oxygen species (ROS), a process tightly connected to and often causative for programmed cell death, is diminished (Fig. 1B).

**Figure 2:**
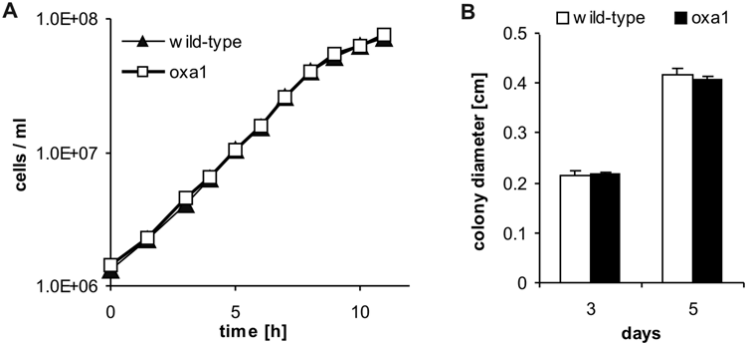
An *oxa1* deletion does not influence the strains growth rate. (A) Growth curves of an *oxa1* deletion strain and the corresponding wild-type strain. Experiment was performed in triplicate. The y-axis is scaled logarithmically. (B) Size of isolated colonies of the indicated strains grown for 3 and 5 days on SCGlu plates, respectively. Diameters were evaluated by processing the photos with Metamorph Imaging (mean±SEM, *n* = 2).

To exclude rho^0^ strain specific effects not accounting for respiration deficiencies, two other respiration impaired strains were investigated. Single gene deletion strains of both, *MGM1* (homolog to human *OPA1*) - a GTPase located in the mitochondrial intermembrane space [24] - and *OXA1* (an insertase of the inner mitochondrial membrane – highly conserved from prokaryotes to mammals [25], [26]) behaved like the (wild-type) rho^0^ strain. After 3 and 5 days, *mgm1* as well as *oxa1* deleted cells retrieved from the whole colony (3 days) or the central region (5 days), and thus mainly older cells, showed significantly increased survival (Fig. 1A) as well as inhibition of ROS generation compared to samples of the wild-type strain (Fig. 1B). Of note, unlike other respiration deficient mutant strains (including rho^0^ and Δ*mgm1*) the Δ*oxa1* strain showed same growth rates as the wild-type strain in liquid cultures and during colony growth, respectively (Fig. 2A and B).

In a complementary approach, seeded colonies (10 and 50 per plate) of wild-type and Δ*oxa1* strains, respectively, were washed from SCGlu plates after 2 and 3 days and clonogenic survival was evaluated. Again, the strain incapable of respiration showed better survival than the respiring wild-type strain (Fig. 1C). Additionally, the survival advantage of the Δ*oxa1* strain occurred already after 2 days, which is in good accordance with previous data that wild-type colonies grown on the usual glucose media where shown to leave the logarithmic growth phase after about 36 hours [27].

To evaluate the induced type of cell death we screened for apoptotic markers using AnnexinV and TUNEL assays. Apoptosis was significantly decreased in a respiration deficient *oxa1* deletion mutant compared to the wild-type control (Fig. 1D). It should be noted that cell wall digestion of wild-type cells was less efficient. Therefore lower amounts of positively stained cells were obtained as compared to the observed cell survival in clonogenic assays after 2 days. This means that the observed suppression of apoptotic markers in the *oxa1* deleted strain is probably even higher than shown in 1D. In this line when treated with 30% more enzyme mix to enhance cell wall digestion, approximately 37% of wild-type cells displayed a TUNEL positive staining (data not shown). Mitochondrial fragmentation is a prerequisite for apoptosis in yeast. Therefore, mitochondrial morphology of cells from wild-type colonies (and not for rho^0^ cells, because they lack respiration) was monitored using dsRED carrying a mitochondrial localization sequence. After 5 days, cells from the central region (thus older cells) showed a drastically increased fragmentation of mitochondria and more punctured structures whereas freshly nourished younger cells from the outer rim displayed a normal tubular structure of mitochondria (Fig 1E).

We conclude that abrogation of respiration suppresses apoptosis and ROS production in a strongly proliferating cell population on solid media.

### Forced enhancement of respiration triggers cell death during seeding of a cell population

**Figure 3:**
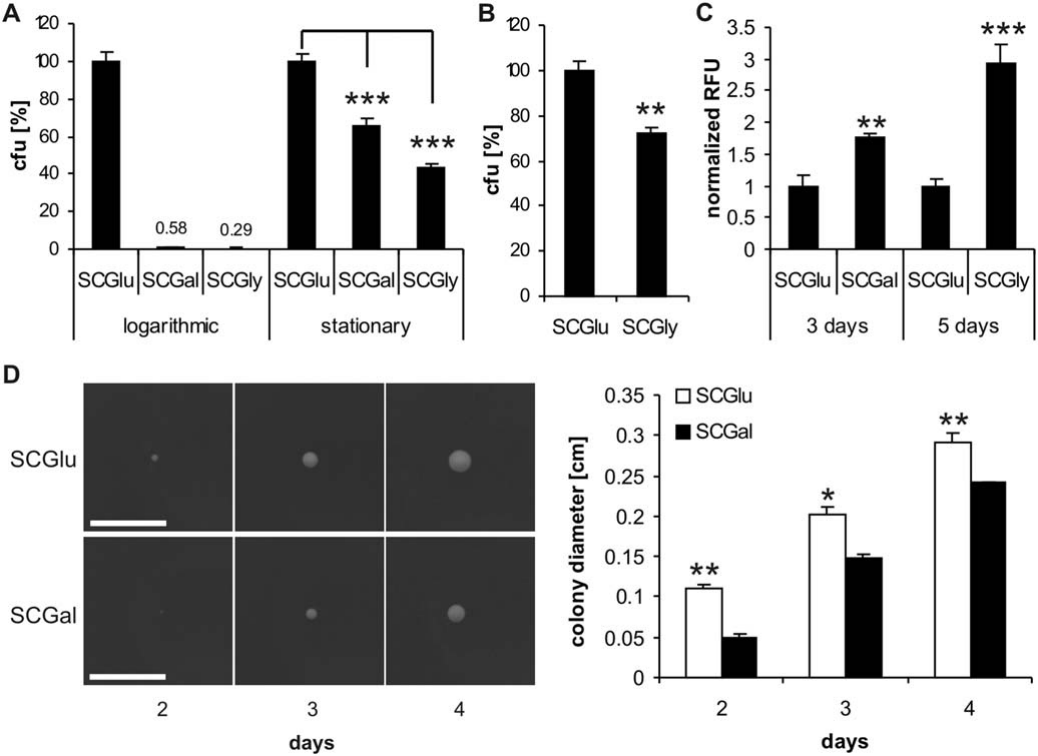
Forced enhancement of respiration triggers cell death, suppresses growth, and elevates ROS production. (A) Clonogenic assay of cultures pre-grown in liquid SCGlu. 500 cells were plated on SCGlu, SCGal, and SCGly agar plates, respectively (mean±SEM, *n* = 3; ***p<0.001). (B) Clonogenic assay of cultures pre-grown in liquid SCGly. 500 cells were plated on SCGly and SCGlu media plates, respectively (mean±SEM, *n* = 2; **p<0.01). (C) Cells from the whole colony (3 days) as well as from the central region (5 days) were stained for reactive oxygen species (ROS) with dihydroethidium (DHE). Values of relative fluorescent units (RFU) were normalized to wild-type strain values (mean±SEM, *n* = 2; **p<0.01, ***p<0.001). (D) Size of isolated wild-type colonies grown on SCGlu and SCGal media plates were monitored by taking photos at indicated time-points. Diameters were evaluated by processing the photos with Metamorph Imaging (white bar represents 1 cm) (mean±SEM, *n* = 3; *p<0.05, **p<0.01).

Novel cancer therapies are based on the assumption that reconstitution or forced enhancement of respiration within solid tumors may lead to growth inhibition and cell death [10], [28], [29]. To test whether genetic enforcement of respiration influences cell death during seeding of a cell population (which is the chronological step before a colony establishes), media-shift experiments using glucose (SCGlu), galactose (SCGal), and glycerol (SCGly) media as alternating sole carbon sources were performed. Thereby, respiration is either suppressed (SCGlu), cooperatively active with fermentation (SCGal), or it represents the exclusive energy source (SCGly). When stationary (non proliferating) liquid populations of yeast wild-type cells grown on SCGlu were shifted to solid SCGal or SCGly, only 65% or 44%, respectively, of seeded cells were able to form a colony (Fig. 3A). Using highly proliferative cultures of wild-type BY4741 strain (from the exponential growth phase) this effect was even more pronounced with nearly no survival on both respiratory media (Fig. 3A). Comparable results were obtained with highly proliferative cultures of other wild-type strains such as AH22, TB50, and DBY746 (data not shown) under our conditions. Even a transfer in the same high respiratory media (from liquid SCGly to solid SCGly) led to a diminished colony formation of ∼70% (compared to a shift from liquid SCGly to solid SCGlu), underlining that specifically the initiation of colony growth is negatively affected by enhanced respiration (Fig. 3B).

### Forced enhancement of respiration suppresses growth and increases ROS production of colonies

**Figure 4:**
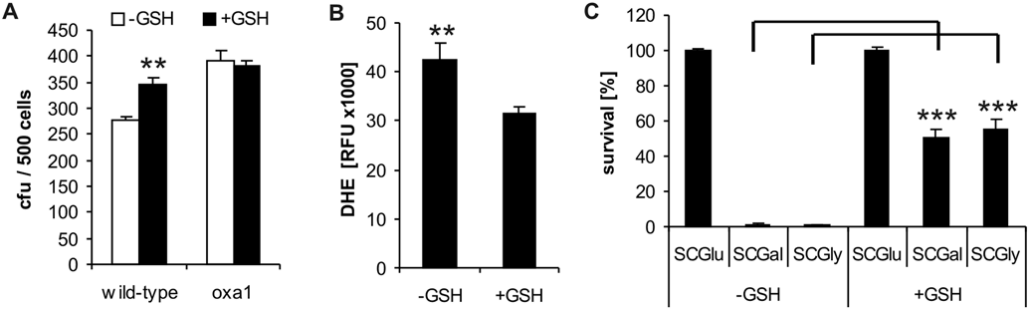
Administration of GSH diminishes ROS levels and enhances survival within wild-type colonies, as well as during seeding on highly respiratory media. (A) Approximately 100 colonies were grown for 36 hours on SCGlu plates with or without 5 mM GSH, washed off and clonogenic assays were performed with the collected cells (mean±SEM, *n* = 3; **p<0.01). Additionally equal amounts of cells were stained for reactive oxygen species (ROS) with dihydroethidium (DHE) and quantified using a fluorescence microplate reader (Genios-Pro). Values of relative fluorescent units (RFU) are displayed (mean±SEM, *n* = 3; **p<0.01) (B). (C) Clonogenic assay of cultures pre-grown in liquid SCGlu. 500 cells were plated on SCGlu, SCGal, or SCGly agar plates, respectively, with or without 1 mM GSH (mean±SEM, *n* = 2; ***p<0.001).

The observed effects of suppressed colony seeding when respiration is high were also reflected in the further growth of the cell population: After two days of growth on solid SCGal, the colonies displayed only half the size of those grown on solid SCGlu, though further size increase (after the initial delay) is comparable (Fig. 3D). The initial delay could be associated with an immediate ROS burst, as further underlined by drastically elevated ROS levels in colonies grown on SCGal or SCGly (Fig. 3C, also see below and Fig. 4C).

Thus, establishing a strongly proliferating solid cell population requires respiration to be repressed.

### Respiration mediated cell death in colonies and newly seeded cells is efficiently suppressed via ROS scavenging

To further prove that respiration and accompanying ROS production is a major cause of apoptosis during colony development we used reduced glutathione (GSH) as a scavenging reagent: Colonies of wild-type and *oxa1* deletion strains were grown on SCGlu agar plates with or without GSH and cell survival was determined after 36 hours in a clonogenic assay. Whereas the *oxa1* deletion strain showed no altered survival upon addition of GSH, survival of wild-type cells was drastically enhanced (Fig 4A). This was accompanied by significantly diminished ROS levels (Fig 4B). Interestingly, extending the fermentative growth phase by doubling glucose concentration in the media plates led to slightly enhanced cell survival (data not shown).

Additionally, the effect of glutathione as ROS scavenger was tested in a shift assay, where highly proliferative wild-type cells pregrown in liquid SCGlu media were seeded onto SCGal and SCGly plates with or without GSH. Strikingly, survival was enhanced to 50% and 55%, respectively, compared to <1% survival on plates without GSH (Fig. 4C). A similar shift of stationary cells yielded an increased survival from 50% and 60% to 75% and 77%, respectively, on GSH containing plates (data not shown). We conclude that scavenging ROS via glutathione enhances cell survival during colony development as well as during seeding on highly respiratory media. Intriguingly, it has been reported recently that in cancer cells and in neurons (that also display the Warburg effect), cytochrome *c* is reduced and held inactive by GSH [30].

So far, research on the Warburg effect has mainly focused on the high glycolytic flux while the accompanying repression of mitochondrial respiration was often left unattended [8], [9]. Our data demonstrate that respiration triggers apoptosis during seeding and development of a cell population, providing direct experimental evidence in support of the Warburg hypothesis during initiation of tumorigenesis. The strongly decreased respiratory capacity may be crucial for tumor malignancy [12] and for resistance against inevitable fluctuating oxygenation in tumors [31], [32]. Interestingly, it has been demonstrated that yeast rho^0^ strains exhibit increased resistance towards certain external apoptotic stimuli such as acetic acid, which triggers death via the mitochondrial pathway [15], [17]. A similar resistance against cell death may also play a role for tumor cells in an acidic surrounding. We speculate that reprogramming of cancer cells follows an ancient mechanism present in *S. cerevisiae* wild-type colonies: high glycolysis in the presence of oxygen.

## Materials and Methods

### Strains and media

Experiments were carried out in BY4741 (MATa *his3Δ1 leu2Δ0 met15Δ0 ura3Δ0*) and respective null mutants of *mgm1* and *oxa1*, respectively, obtained from Euroscarf. Strain BY4741 rho^0^ was constructed by several rounds of ethidiumbromide treatment as previously described [33]. To perform fluorescence microscopy of mitochondrial structures the plasmid dsRED-MLS (dsRED carrying a mitochondrial localization sequence) [34] was transformed in strain BY4741. For survival platings YEPD (1% yeast extract, 2% peptone, and 2% glucose) media was supplemented with 2% agar. For all assays, strains were grown in SC medium containing 0.17% yeast nitrogen base (Difco), 0.5% (NH_4_)_2_SO_4_, and 30 mg/l of all amino acids (except 80 mg/l histidine, 200 mg/l leucine (without leucine when working with plasmid dsRED-MLS)), 30 mg/l adenine, and 320 mg/l uracil with 2% glucose (SCGlu), 2% galactose (SCGal), and 3% glycerol (SCGly), respectively, supplemented with 2% agar, where needed. Reduced glutathione (GSH; Sigma-Aldrich) was added at concentrations of 1 or 5 mM, where needed.

### Isolated monocolony assay

Six centimeter petridishes, supplied with 13 ml of solid SC media (with or without leucine) were used. Single cells of 16 to 20 hours overnight cultures were placed onto solid SC media plates using a micromanipulator (Series 200, Singer Instruments). To minimize dehydration of the solid plates, incubation was performed at 28°C in a closed, humidified breeding chamber (KBF115, Binder). For diameter determination, photos were taken at the indicated time-points (Microbiology-Colony-Counter, Lemnatec) and processed using Metamorph imaging.

### Survival plating, media-shift experiments, and growth curves

To determine survival of cells within the colony, either whole isolated colonies (in the case of 3 days) and samples from the central regions of the isolated colonies (in the case of 5 days) were removed at indicated time-points, or SCGlu plates with approximately 100 colonies (after 36 hours), 50 colonies (after 2 days) or 10 colonies (after 3 days) were washed off. Cell counts were determined by a CASY cell counting device (Schärfe Systems) and 500 cells were plated on YEPD agar plates. Colony forming units (cfu) were determined after 2 or 3 days (in the case of respiration deficient strains rho^0^ and Δ*mgm1*) with a Microbiology-Colony-Counter (Lemnatec) and processed using SAWmicrobio version 3.1.

For media-shift experiments, cultures were grown in SCGlu and SCGly liquid media to logarithmic (6 h) and stationary (24 h) phase, respectively, and 500 cells were plated on respective solid media plates as well as on SCGlu plates in any case.

For assessment of growth rates in liquid media, overnight cultures were grown in SCGlu. Cultures were inoculated to 5×10^5^ cells in SCGlu and cell growth was monitored over 12 hours using a CASY cell counter device.

### Staining for apoptotic markers and fluorescence microscopy

Staining of reactive oxygen species (ROS) with dihydroethidium (DHE), Annexin V-staining, and TUNEL-staining were performed as described previously [34]. In each sample 30.000 cells were evaluated using flow cytometry (FACS-Aria, BD) and processed using FACSDiva software. Alternatively equal amounts of DHE stained cells were measured in a microplate fluorescence reader (Genios-Pro, Tecan).

Mitochondrial morphology was inspected by fluorescence microscopy using a dsRED filter on a Zeiss Axioskop microscope. Cells of the central as well as the outer region of isolated monocolonies of wild-type cells harbouring plasmid dsRED-MLS were monitored after 5 days.

### Statistical analysis

All statistical analyses were performed using Students T-Test (one-tailed, unpaired), with *p<0.05, **p<0.01 and ***p<0.001, respectively.
